# In This Issue

**Published:** 2006

## INTRODUCTION TO THE NATIONAL EPIDEMIOLOGIC SURVEY ON ALCOHOL AND RELATED CONDITIONS

In 2001–2002, the National Institute on Alcohol Abuse and Alcoholism conducted the first wave of the National Epidemiologic Survey on Alcohol and Related Conditions (NESARC), the largest and most ambitious comorbidity study ever undertaken. The survey included a battery of questions about present and past alcohol consumption, alcohol use disorders (AUDs), co-occurring mental health disorders, and the use of alcoholism treatment services. NESARC also included questions related to tobacco and illicit drug use. Its unprecedented sample size (*n* = 43,093) made it possible to obtain data even on rare conditions. And it included data from populations not typically captured by national surveys, including Blacks, Hispanics, people living in Hawaii and Alaska, and people living in residences such as boarding houses and campus housing. In this introductory article, Drs. Bridget F. Grant and Deborah A. Dawson briefly describe the purpose and design of NESARC and summarize some of the major conclusions that have been drawn from its data to date. For example, researchers have been able to determine the prevalence of alcohol abuse and dependence as well as of the comorbidity between AUDs and other drug use disorders, mood and anxiety disorders, and personality disorders. Other analyses have yielded information on recovery from AUDs and on the prevalence of driving after drinking. Analyses using the NESARC data set have only just begun, but these early studies provide insight into the range and dimensions of this dataset and some of the important issues that can be studied using this information. (pp. 74–78)

## THE 12-MONTH PREVALENCE AND TRENDS IN DSM–IV ALCOHOL ABUSE AND DEPENDENCE: UNITED STATES, 1991–1992 AND 2001–2002

Alcohol abuse and dependence are among the most prevalent mental disorders in the United States, yet relatively little information has been available on the actual prevalence of these alcohol use disorders and on changes in prevalence over time. To close this knowledge gap, Drs. Bridget F. Grant, Deborah A. Dawson, Frederick S. Stinson, S. Patricia Chou, and Mary C. Dufour, as well as Mr. Roger P. Pickering analyzed data from two large national surveys sponsored by the National Institute on Alcohol Abuse and Alcoholism: the 2001–2002 National Epidemiologic Survey on Alcohol and Related Conditions (NESARC) and the 1991–1992 National Longitudinal Alcohol Epidemiologic Survey. These analyses found that the prevalence of both alcohol abuse and dependence varies substantially with age, gender, and race/ethnicity. For example, in 2001–2002, the prevalence of alcohol abuse was significantly higher among Whites than among Blacks, Asians, and Hispanics, whereas the prevalence of dependence was significantly higher among Whites, Native Americans, and Hispanics than among Asians. In all population subgroups, rates of alcohol abuse and dependence were highest among men under age 30. Changes in the prevalence of alcohol abuse and dependence between 1991–1992 and 2001–2002 also differed among population subgroups. For example, whereas the prevalence of dependence overall declined over that period, it increased significantly among young Asian men and young Black women. As the authors explain, monitoring these trends is important for designing appropriate prevention and intervention programs. (pp. 79–91)

## COMORBIDITY BETWEEN DSM–IV ALCOHOL AND SPECIFIC DRUG USE DISORDERS IN THE UNITED STATES: RESULTS FROM THE NATIONAL EPIDEMIOLOGIC SURVEY ON ALCOHOL AND RELATED CONDITIONS

Many people with alcohol abuse and dependence also suffer from other drug use disorders, exacerbating the harmful consequences of the alcohol use disorders (AUDs). However, few national surveys have determined the prevalence of comorbidity between AUDs and other drug use disorders. To obtain more accurate estimates of the prevalence of comorbidity and of factors influencing the risk of comorbidity, Drs. Frederick S. Stinson, Bridget F. Grant, Deborah A. Dawson, Boji Huang, and Tulshi Saha, as well as Ms. W. June Ruan assessed data from the 2001–2002 National Epidemiologic Survey on Alcohol and Related Conditions. These analyses demonstrated that about one-eighth of all people with an AUD also had a comorbid drug use disorder. The prevalence of comorbid alcohol and drug use disorders was particularly high in people who were young, male, never married, and/or of low socioeconomic status. Investigating the potential impact of comorbid alcohol and drug use disorders on treatment-seeking, the investigators also found that people with comorbid disorders were three times as likely to seek treatment as those with AUDs only, highlighting the need to integrate alcoholism and drug treatment services. (pp. 94–106)

## PREVALENCE AND COOCCURRENCE OF SUBSTANCE USE DISORDERS AND INDEPENDENT MOOD AND ANXIETY DISORDERS: RESULTS FROM THE NATIONAL EPIDEMIOLOGIC SURVEY ON ALCOHOL AND RELATED CONDITIONS

Many people with an alcohol or drug use disorder also are diagnosed with a mood or anxiety disorder at some time in their lives. It is often difficult to determine whether these mood or anxiety disorders arise independently or are drug induced; however, this distinction has important treatment implications. To clarify the relationship between alcohol or drug use disorders on the one hand and mood or anxiety disorders on the other hand, Drs. Bridget F. Grant, Frederick S. Stinson, Deborah A. Dawson, S. Patricia Chou, Mary C. Dufour, and Wilson Compton as well as Mr. Roger P. Pickering and Mr. Kenneth Kaplan analyzed data obtained in the 2001–2002 National Epidemiologic Survey on Alcohol and Related Conditions. The study found that compared with the general population, the prevalence of mood and anxiety disorders was substantially higher in patients with an alcohol or drug use disorder. Furthermore, the rates of co-occurring alcohol or drug use disorders and mood or anxiety disorders were even higher in people seeking treatment for either of these conditions. The investigators also found that only in a small fraction of cases was the mood or anxiety disorder drug induced; in all other cases, it arose independently of the alcohol or drug use disorder. These findings indicate that patients should be assessed for the presence of both alcohol or drug use disorders and mood or anxiety disorders and that treatment must address both types of psychiatric disorders. (pp. 107–120)

## CO-OCCURRENCE OF 12-MONTH ALCOHOL AND DRUG USE DISORDERS AND PERSONALITY DISORDERS IN THE UNITED STATES: RESULTS FROM THE NATIONAL EPIDEMIOLOGIC SURVEY ON ALCOHOL AND RELATED CONDITIONS

Personality disorders, particularly antisocial personality disorder, commonly have been found in people undergoing treatment for alcohol or other drug abuse or dependence. Existing analyses of these associations, however, have had serious limitations (e.g., no distinction between alcohol and other drug use disorders, focus primarily on males, focus on patients in treatment). To address these limitations, Drs. Bridget F. Grant, Frederick S. Stinson, Deborah A. Dawson, and S. Patricia Chou as well as Ms. June Ruan and Mr. Roger P. Pickering examined data obtained in the 2001–2002 National Epidemiologic Survey on Alcohol and Related Conditions for the prevalence of cooccurrence of alcohol and other drug use disorders with personality disorders. These analyses found that more than one-fourth of people with an alcohol use disorder and almost one-half of people with another drug use disorder had at least one co-occurring personality disorder. Similarly, alcohol or other drug use disorders were found in a substantial proportion of people with personality disorders. Further analyses of the association between specific personality disorders and alcohol and other drug use disorders determined that both alcohol and other drug use disorders were particularly highly associated with antisocial, histrionic, and dependent personality disorders. Moreover, gender differences existed in the associations between specific personality disorders and alcohol and other drug use disorders. These observations underscore the importance of taking comorbid personality disorders into account when designing treatment programs for patients with alcohol and other drug use disorders. (pp. 121–130)

## RECOVERY FROM DSM–IV ALCOHOL DEPENDENCE: UNITED STATES, 2001–2002

Studies have shown that many people can recover from alcohol abuse or dependence, both with and without participating in formal treatment or self-help programs. Not all of these people remain abstinent, and many continue to drink at varying levels. To further explore the prevalence and types of recovery from alcohol dependence, Drs. Deborah A. Dawson, Bridget F. Grant, Frederick S. Stinson, Patricia S. Chou, and Boji Huang as well as Ms. W. June Ruan analyzed data from the 2001–2002 National Epidemiologic Survey on Alcohol and Related Conditions to determine the drinking status of people diagnosed with alcohol dependence prior to the year preceding the survey interview. This analysis determined that although only about 25 percent of these people had received any treatment, only about one-quarter of them were still classified as dependent, whereas more than one-third were abstinent or considered low-risk drinkers. The rest were classified either as being in partial remission or as drinking at levels putting them at risk of relapse. Of the abstinent or low-risk drinkers, the vast majority had been in remission for at least 5 years, indicating stable recovery. Several factors can influence the likelihood of recovery, including marital status, age, gender, severity of dependence, presence of personality disorders, or treatment history. Knowledge of such factors may help improve existing treatment approaches. (pp. 131–142)

## TWELVE-MONTH PREVALENCE AND CHANGES IN DRIVING AFTER DRINKING: UNITED STATES, 1991–1992 AND 2001–2002

One of the most common, and potentially most harmful, sequelae of alcohol consumption is driving after drinking, which in the United States accounts for about 40 percent of all motor vehicle fatalities. To determine the prevalence of driving after drinking, as well as changes in prevalence over the past decade, Drs. S. Patricia Chou, Bridget F. Grant, Deborah A. Dawson, Frederick S. Stinson, Tulshi Saha, and Mr. Roger P. Pickering analyzed data from the 2001–2002 National Epidemiologic Survey on Alcohol and Related Conditions and compared them with data from the 1991–1992 National Longitudinal Alcohol Epidemiologic Survey. The investigators found that in 2001–2002, about 6 million American adults drove after drinking, a reduction by about one-quarter from a decade earlier. Young adults were most likely to drive after drinking, particularly young adult men. However, this age group also showed the greatest decline in driving after drinking over the past decade. Finally, although in general, males’ and females’ rates of drinking and driving became more similar, the gender difference in drinking-driving rates among 18- to 20-year-olds became more pronounced, reflecting a large decline in drinking and driving among women in this age group. (pp. 143–151)

## COMMENTARY: NESARC FINDINGS ON ALCOHOL ABUSE AND DEPENDENCE

As described in the articles in this journal issue, the National Epidemiological Study on Alcohol and Related Conditions (NESARC) has generated vast amounts of information on alcohol use disorders (AUDs) in the general population of the United States, their co-occurrence with numerous other conditions, and a variety of other related issues. But what is the clinical relevance of this wealth of information? That is, what do the findings from NESARC mean for people working in the prevention and treatment fields? As Dr. Raul Caetano explains, the NESARC data have many practical implications. For example, data on the prevalence of AUDs in various population subgroups provide treatment programs with information on the characteristics of the expected patient for whom they should tailor interventions. Similarly, information on the comorbidity of AUDs with other psychiatric disorders is relevant for designing successful prevention approaches, because prevention strategies addressing only one problem may not be as effective as those targeting comorbid conditions. Finally, NESARC results can provide guidance for planning treatment strategies and designing provider training programs. For example, based on the NESARC findings, it appears essential that alcoholism treatment providers be trained to identify the signs and symptoms of common comorbid conditions and to make appropriate referrals for patients with AUDs and comorbid conditions. As these early studies show, NESARC findings do indeed have practical implications and will help clinicians to better target their prevention and intervention measures. (pp. 152–155)

